# Pointwise approximation of modified conjugate functions by matrix operators of conjugate Fourier series of $2\pi /r$-periodic functions

**DOI:** 10.1186/s13660-018-1684-0

**Published:** 2018-04-20

**Authors:** Mateusz Kubiak, Włodzimierz Łenski, Bogdan Szal

**Affiliations:** Faculty of Mathematics, Computer Science and Econometrics, University of Zielona Góra, Zielona Góra, Poland

**Keywords:** 42A24, Rate of approximation, Summability of Fourier series

## Abstract

We extend the results of Xh. Z. Krasniqi (Acta Comment. Univ. Tartu Math. 17:89–101, 2013) and the authors (Acta Comment. Univ. Tartu Math. 13:11–24, 2009; Proc. Est. Acad. Sci. 67:50–60, 2018) to the case when considered function is $2\pi/r$-periodic and the measure of approximation depends on *r*-differences of the entries of the considered matrices.

## Introduction

Let $L_{2\pi/r}^{p}\ (1\leq p<\infty)$ be the class of all $2\pi /r$-periodic real-valued functions, integrable in the Lebesgue sense with the *p*th power over $Q_{r}=$ $[-\pi/r,\pi/r]$ with the norm

$$ \Vert f \Vert _{L_{2\pi/r}^{p}}= \bigl\Vert f ( \cdot) \bigr\Vert _{L_{2\pi /r}^{p}}:= \biggl( \int_{Q_{r}}\bigl\vert f(t)\bigr\vert ^{p}\,dt \biggr) ^{1/p}, $$

where $r\in \mathbb{N} $. It is clear that $L_{2\pi/r}^{p}\subset L_{2\pi/1}^{p}=L_{2\pi}^{p}$ and for $f\in L_{2\pi/r}^{p}$

$$ \Vert f \Vert _{L_{2\pi}^{p}}=r^{1/p} \Vert f \Vert _{L_{2\pi/r}^{p}}. $$

Taking into account the above relations, we will consider, for $f\in L_{2\pi /r}^{1}$, the trigonometric Fourier series as such a series of $f\in L_{2\pi }^{1}$ in the following form:

$$ Sf(x):=\frac{a_{0}(f)}{2}+\sum_{\nu=1}^{\infty} \bigl(a_{\nu}(f)\cos\nu x+b_{\nu}(f)\sin\nu x \bigr) $$

with the partial sums $S_{k}f$ and the conjugate one

$$ \widetilde{S}f(x):=\sum_{\nu=1}^{\infty} \bigl(a_{\nu}(f)\sin\nu x-b_{\nu }(f)\cos\nu x \bigr) $$

with the partial sums $\widetilde{S}_{k}f$. We also know that if $f\in L_{2\pi}^{1}$, then

$$ \widetilde{f} ( x ) :=-\frac{1}{\pi} \int_{0}^{\pi}\psi_{x} ( t ) \frac{1}{2}\cot\frac{t}{2}\,dt=\lim_{\epsilon\rightarrow0^{+}}\widetilde{f} ( x,\epsilon) =\lim_{\epsilon\rightarrow0^{+}}\widetilde{f}_{r} ( x,\epsilon) , $$

where, for $r\in \mathbb{N} $,

$$ \widetilde{f}_{r} ( x,\epsilon) := \textstyle\begin{cases} -\frac{1}{\pi} ( \sum_{m=0}^{ [ r/2 ] -1}\int_{\frac{2m\pi}{r}+\epsilon}^{\frac{2 ( m+1 ) \pi}{r}-\epsilon}+\int_{\frac{2 [ r/2 ] \pi}{r}+\epsilon}^{\frac{ ( 2 [ r/2 ] +1 ) \pi}{r}} ) \psi_{x} ( t ) \frac{1}{2}\cot\frac{t}{2}\,dt&\text{for an odd }r, \\ -\frac{1}{\pi}\sum_{m=0}^{ [ r/2 ] -1}\int_{\frac{2m\pi}{r}+\epsilon}^{\frac{2 ( m+1 ) \pi}{r}-\epsilon}\psi_{x} ( t ) \frac{1}{2}\cot\frac{t}{2}\,dt&\text{for an even }r,\end{cases} $$

and

$$ \widetilde{f} ( x,\epsilon) =\widetilde{f}_{1} ( x,\epsilon) :=- \frac{1}{\pi} \int_{\epsilon}^{\pi}\psi_{x} ( t ) \frac{1}{2}\cot\frac{t}{2}\,dt, $$

with

$$ \psi_{x} ( t ) :=f ( x+t ) -f ( x-t ) , $$

exist for almost all *x* (cf. [4, Th. (3.1) IV]).

Let $A:= ( a_{n,k} ) $ be an infinite matrix of real numbers such that

$$ a_{n,k}\geq0\quad\text{when }k,n=0,1,2,\ldots, \qquad\lim _{n\rightarrow \infty}a_{n,k}=0\quad\text{and}\quad\sum _{k=0}^{\infty}a_{n,k}=1, $$

but $A^{\circ}:= ( a_{n,k} ) _{k=0}^{n}$, where

$$ a_{n,k}=0\quad\text{when }k>n. $$

We will use the notations

$$ A_{n,r}=\sum_{k=0}^{\infty} \vert a_{n,k}-a_{n,k+r} \vert ,\qquad A_{n,r}^{\circ}= \sum_{k=0}^{n} \vert a_{n,k}-a_{n,k+r} \vert \text{ } $$

for $r\in \mathbb{N} $ and

$$ \widetilde{T}_{n,A} f ( x ) :=\sum _{k=0}^{\infty}a_{n,k}\widetilde{S}_{k}f ( x ) \quad ( n=0,1,2,\ldots ) $$

for the *A*-transformation of *S̃f*.

In this paper, we will study the estimate of $\vert \widetilde{T}_{n,A} f ( x ) -\widetilde{f}_{r} ( x,\epsilon ) \vert $ by the function of modulus of continuity type, i.e. a nondecreasing continuous function *ω̃* having the following properties: $\widetilde{\omega} ( 0 ) =0$, $\widetilde{\omega}( \delta_{1}+\delta_{2} ) \leq\widetilde{\omega} ( \delta _{1} ) +\widetilde{\omega} ( \delta_{2} ) $ for any $0\leq \delta_{1}\leq\delta_{2}\leq\delta_{1}+\delta_{2}\leq2\pi$. We will also consider functions from the subclass $L_{2\pi/r}^{p} ( \widetilde{\omega} ) _{\beta}$ of $L_{2\pi/r}^{p}$ for $r\in\mathbb{ N}$:

$$ L_{2\pi/r}^{p} ( \widetilde{\omega} ) _{\beta}= \bigl\{ f \in L_{2\pi/r}^{p}:\widetilde{\omega}_{\beta} ( f,\delta ) _{L_{2\pi/r}^{p}}=O \bigl( \widetilde{\omega} ( \delta) \bigr) \text{ when } \delta\in[ 0,2\pi] \text{ and }\beta\geq0 \bigr\} , $$

where

$$ \widetilde{\omega}_{\beta}f ( \delta) _{L_{2\pi /r}^{p}}=\sup _{0\leq \vert t \vert \leq\delta} \biggl\{ \biggl\vert \sin \frac{rt}{2} \biggr\vert ^{\beta} \bigl\Vert \psi_{\cdot} ( t ) \bigr\Vert _{L_{2\pi/r}^{p}} \biggr\} . $$

It is easy to see that $\widetilde{\omega}_{0}f ( \cdot ) _{L_{2\pi/r}^{p}}=\widetilde{\omega}f ( \cdot ) _{L_{2\pi /r}^{p}}$ is the classical modulus of continuity. Moreover, it is clear that for $\beta\geq\alpha\geq0$

$$ \widetilde{\omega}_{\beta}f ( \delta) _{L_{2\pi/r}^{p}}\leq \widetilde{ \omega}_{\alpha}f ( \delta) _{L_{2\pi/r}^{p}} $$

and consequently

$$ L_{2\pi/r}^{p} ( \widetilde{\omega} ) _{\alpha}\subseteq L_{2\pi /r}^{p} ( \widetilde{\omega} ) _{\beta}. $$

The deviation $\widetilde{T}_{n,A} f ( x ) -\widetilde{f}_{r} ( x,\epsilon ) $ was estimated with $r=1$ in [2] and generalized in [1] as follows:

### Theorem A

([1, Theorem 8, p. 95])

*If* $f\in L_{2\pi }^{p} ( \widetilde{\omega} ) _{\beta}$ *with* $1< p<\infty $ *and* $0\leq\beta<1-\frac{1}{p}$, *where* *ω̃* *satisfies the conditions*:

1 $$ \biggl\{ \int_{\frac{\pi}{n+1} }^{\pi} \biggl( \frac{t^{-\gamma} \vert \psi_{x} ( t ) \vert }{\widetilde{\omega} ( t ) } \biggr) ^{p}\sin^{\beta p}\frac{t}{2}\,dt \biggr\} ^{1/p}=O_{x} \bigl( ( n+1 ) ^{\gamma} \bigr) $$

*with* $0<\gamma<\beta+\frac{1}{p}$ *and*

2 $$ \biggl\{ \int_{0}^{\frac{\pi}{n+1}} \biggl( \frac{t \vert \psi_{x} ( t ) \vert }{\widetilde{\omega} ( t ) } \biggr) ^{p}\sin^{\beta p}\frac{t}{2}\,dt \biggr\} ^{1/p}=O_{x} \bigl( ( n+1 ) ^{-1} \bigr) , $$

*then*

$$ \biggl\vert \widetilde{T}_{n,A^{\circ}} f ( x ) -\widetilde{f} \biggl( x,\frac{\pi}{n+1} \biggr) \biggr\vert =O_{x} \biggl( ( n+1 ) ^{\beta+\frac{1}{p}+1}A_{n,1}^{\circ}\widetilde{\omega } \biggl( \frac{\pi}{n+1} \biggr) \biggr) . $$

The next essential generalizations and improvements in [3, Theorem 1] were given. In these results $\widetilde{f}_{r} ( x,\epsilon ) $ and $A_{n,r}$ (with $r\in \mathbb{N} $) instead of $\widetilde{f}_{1} ( x,\epsilon ) =\widetilde{f}( x,\epsilon ) $ and $A_{n,1}^{\circ}$, respectively, were taken. We can formulate them as follows.

### Theorem B

([3, Theorem 1])

*If* $f\in L_{2\pi }^{p}$, $1< p<\infty$, $0\leq\beta<1-\frac{1}{p}$ *and a function* *ω̃* *of modulus of continuity type satisfies the conditions*:

3 $$ \biggl\{ \int_{0}^{\frac{\pi}{r ( n+1 ) }} \biggl( \frac{t \vert \psi_{x} ( t ) \vert \vert \sin\frac{rt}{2} \vert ^{\beta}}{\widetilde{\omega} ( t ) } \biggr) ^{p}\,dt \biggr\} ^{1/p}=O_{x} \bigl( ( n+1 ) ^{-1} \bigr) $$

*for* $r\in \mathbb{N} $,

4 $$ \biggl\{ \int_{\frac{2m\pi}{r}}^{\frac{2m\pi}{r}+\frac{\pi}{r ( n+1 ) }} \biggl( \frac{ \vert \psi_{x} ( t ) \vert \vert \sin\frac{rt}{2} \vert ^{\beta}}{\widetilde{\omega} ( t-\frac{2m\pi}{r} ) } \biggr) ^{p}\,dt \biggr\} ^{1/p}=O_{x} ( 1 ) $$

*for a natural* $r\geq3$, *where* $m\in \{ 1,\ldots [ \frac{r}{2} ] \} $ *when* *r* *is an odd or* $m\in \{ 1,\ldots [ \frac{r}{2} ] -1 \} $ *when* *r* *is an even natural number*, *and*

5 $$ \biggl\{ \int_{\frac{2m\pi}{r}+\frac{\pi}{r ( n+1 ) }}^{\frac{2m\pi}{r}+\frac{\pi}{r}} \biggl( \frac{ \vert \psi_{x} ( t ) \vert \vert \sin\frac{rt}{2} \vert ^{\beta}}{\widetilde{\omega} ( t ) ( t-\frac{2m\pi}{r} ) ^{\gamma }} \biggr) ^{p}\,dt \biggr\} ^{1/p}=O_{x} \bigl( ( n+1 ) ^{\gamma } \bigr) , $$

*for* $r\in \mathbb{N} $ *with* $0<\gamma<\beta+\frac{1}{p}$, *where* $m\in \{ 0,\ldots [ \frac{r}{2} ] \} $ *when* *r* *is an odd or* $m\in \{ 0,\ldots [ \frac{r}{2} ] -1 \} $ *when* *r* *is an even natural number*. *Moreover*, *let* *ω̃* *satisfy*, *for a natural* $r\geq 2$, *the conditions*:

6 $$\begin{aligned} &\biggl\{ \int_{\frac{2 ( m+1 ) \pi}{r}-\frac{\pi}{r ( n+1 ) }}^{\frac{2 ( m+1 ) \pi}{r}} \biggl( \frac{ \vert \psi_{x} ( t ) \vert \vert \sin\frac{rt}{2} \vert ^{\beta}}{\widetilde{\omega} ( \frac{2 ( m+1 ) \pi}{r}-t ) } \biggr) ^{p}\,dt \biggr\} ^{1/p}=O_{x} ( 1 ) , \end{aligned}$$

7 $$\begin{aligned} &\biggl\{ \int_{\frac{2 ( m+1 ) \pi}{r}-\frac{\pi}{r}}^{\frac{2 ( m+1 ) \pi}{r}-\frac{\pi}{r ( n+1 ) }} \biggl( \frac{ \vert \psi_{x} ( t ) \vert \vert \sin\frac{rt}{2} \vert ^{\beta}}{\widetilde{\omega} ( t ) ( \frac{2 ( m+1 ) \pi}{r}-t ) ^{\gamma}} \biggr) ^{p}\,dt \biggr\} ^{1/p}=O_{x} \bigl( ( n+1 ) ^{\gamma} \bigr) , \end{aligned}$$

*with* $0<\gamma<\beta+\frac{1}{p}$, *where* $m\in \{ 0,\ldots [ \frac{r}{2} ] -1 \} $. *If a matrix* *A* *is such that*

8 $$ \sum_{k=0}^{\infty} ( k+1 ) ^{2}a_{n,k}=O \bigl( ( n+1 ) ^{2} \bigr) $$

*and*

9 $$ \Biggl[ \sum_{l=0}^{n}\sum _{k=l}^{r+l-1}a_{n,k} \Biggr] ^{-1}=O ( 1 ) $$

*with* $r\in \mathbb{N} $ *are true*, *then*

$$ \biggl\vert \widetilde{T}_{n,A} f ( x ) - \widetilde{f}_{r} \biggl( x,\frac{\pi}{r ( n+1 ) } \biggr) \biggr\vert =O_{x} \biggl( ( n+1 ) ^{\beta+\frac{1}{p}+1}A_{n,r} \widetilde{\omega} \biggl( \frac{\pi}{n+1} \biggr) \biggr) . $$

### Theorem C

([3, Theorem 2])

*Let* $f\in L_{2\pi }^{p}$, $1< p<\infty$, $0\leq\beta<1-\frac{1}{p}$ *and a function* *ω̃* *of modulus of continuity type satisfy*, *for* $r\in \mathbb{N} $, *the conditions*: (4) *and* (5) *with* $0<\gamma<\beta+\frac{1}{p}$, *where* $m\in \{ 0,\ldots [ \frac{r}{2} ] \} $ *when* *r* *is an odd or* $m\in \{ 0,\ldots [ \frac{r}{2} ] -1 \} $ *when* *r* *is an even natural number*. *Moreover*, *let* *ω̃* *satisfy*, *for a natural* $r\geq 2$, *the conditions* (6) *and* (7) *with* $0<\gamma<\beta+\frac{1}{p}$, *where* $m\in \{ 0,\ldots [ \frac{r}{2} ] -1 \} $. *If a matrix* *A* *is such that*

10 $$ \sum_{k=0}^{\infty} ( k+1 ) a_{n,k}=O ( n+1 ) , $$

*and* (9) *with* $r\in \mathbb{N} $ *are true*, *then*

$$ \biggl\vert \widetilde{T}_{n,A} f ( x ) - \widetilde{f}_{r} \biggl( x,\frac{\pi}{r ( n+1 ) } \biggr) \biggr\vert =O_{x} \biggl( ( n+1 ) ^{\beta+\frac{1}{p}+1}A_{n,r} \widetilde{\omega} \biggl( \frac{\pi}{n+1} \biggr) \biggr) . $$

In our theorems we generalize the above results considering $2\pi/r$-periodic functions and using simpler assumptions.

In the paper $\sum_{k=a}^{b}=0$ when $a>b$.

## Statement of the results

To begin with, we will present the estimates of the quantities

$$ \biggl\vert \widetilde{T}_{n,A} f ( x ) - \widetilde{f}_{r} \biggl( x,\frac{\pi}{r ( n+1 ) } \biggr) \biggr\vert \quad\text{and}\quad \biggl\Vert \widetilde{T}_{n,A} f ( \cdot) -\widetilde{f}_{r} \biggl( \cdot, \frac{\pi}{r ( n+1 ) } \biggr) \biggr\Vert _{L_{2\pi/r}^{p}}. $$

Finally, we will formulate some remarks and corollaries.

### Theorem 1

*Suppose that* $f\in L_{2\pi/r}^{p}$, $1< p<\infty$, $r\in\mathbb{N}$, $0\leq\beta<1-\frac{1}{p}$ *and a function* *ω̃* *of the modulus of continuity type satisfies the conditions*:

11 $$ \biggl\{ \int_{0}^{\frac{\pi}{r ( n+1 ) }} \biggl( \frac{t \vert \psi_{x} ( t ) \vert \vert \sin\frac{rt}{2}\vert ^{\beta}}{\widetilde{\omega} ( t ) } \biggr) ^{p}\,dt \biggr\} ^{1/p}=O_{x} \bigl( ( n+1 ) ^{-1} \bigr) , $$

*when* $r=1$ *or*

12 $$ \biggl\{ \int_{0}^{\frac{\pi}{r ( n+1 ) }} \biggl( \frac{\vert \psi_{x} ( t ) \vert \vert \sin\frac{rt}{2}\vert ^{\beta}}{\widetilde{\omega} ( t ) } \biggr) ^{p}\,dt \biggr\} ^{1/p}=O_{x} ( 1 ) , $$

*when* $r\geq2$, *and*

13 $$ \biggl\{ \int_{\frac{\pi}{r ( n+1 ) }}^{\frac{\pi}{r}} \biggl( \frac{ \vert \psi_{x} ( t ) \vert \vert \sin\frac{rt}{2} \vert ^{\beta}}{\widetilde{\omega} ( t ) t^{\gamma}} \biggr) ^{p}\,dt \biggr\} ^{1/p}=O_{x} \bigl( ( n+1 ) ^{\gamma} \bigr) , $$

*for* $r\in \mathbb{N} $ *with* $0<\gamma<\beta+\frac{1}{p}$. *If a matrix* *A* *is such that* (8) *and* (9) *are true*, *then*

$$ \biggl\vert \widetilde{T}_{n,A} f ( x ) - \widetilde{f}_{r} \biggl( x,\frac{\pi}{r ( n+1 ) } \biggr) \biggr\vert =O_{x} \biggl( ( n+1 ) ^{\beta+\frac{1}{p}+1}A_{n,r} \widetilde{\omega} \biggl( \frac{\pi}{n+1} \biggr) \biggr) . $$

### Theorem 2

*Suppose that* $f\in L_{2\pi/r}^{p}$, $1< p<\infty$, $r\in\mathbb{N}$, $0\leq\beta<1-\frac{1}{p}$ *and a function* *ω̃* *of the modulus of continuity type satisfies the conditions* (12) *and* (13) *for* $r\in \mathbb{N} $ *with* $0<\gamma<\beta+\frac{1}{p}$. *If a matrix* *A* *is such that* (10) *and* (9) *are true*, *then*

$$ \biggl\vert \widetilde{T}_{n,A} f ( x ) - \widetilde{f}_{r} \biggl( x,\frac{\pi}{r ( n+1 ) } \biggr) \biggr\vert =O_{x} \biggl( ( n+1 ) ^{\beta+\frac{1}{p}+1}A_{n,r} \widetilde{\omega} \biggl( \frac{\pi}{n+1} \biggr) \biggr) . $$

### Remark 1

The Hölder inequality gives

$$\begin{aligned} \sum_{k=0}^{\infty} ( k+1 ) a_{n,k} &=\sum_{k=0}^{\infty} ( k+1 ) a_{n,k}^{1/2}a_{n,k}^{1/2}\leq \Biggl[ \sum _{k=0}^{\infty} ( k+1 ) ^{2}a_{n,k} \Biggr] ^{1/2} \Biggl[ \sum_{k=0}^{\infty}a_{n,k} \Biggr] ^{1/2} \\ &= \Biggl[ \sum_{k=0}^{\infty} ( k+1 ) ^{2}a_{n,k} \Biggr] ^{1/2} \end{aligned}$$

and thus the condition (8) implies (10), but the condition (12) implies (11). Therefore Theorems 1 and 2 are not comparable.

### Theorem 3

*Let* $f\in L_{2\pi/r}^{p} ( \widetilde{\omega} ) _{\beta}$, $1< p<\infty$, $r\in\mathbb{N}$ *and* $0\leq\beta<1-\frac{1}{p}$. *If a matrix* *A* *is such that* (9) *and* (8) *or* (10) *are true*, *then*

$$ \biggl\Vert \widetilde{T}_{n,A} f ( \cdot) - \widetilde{f}_{r} \biggl( \cdot,\frac{\pi}{r ( n+1 ) } \biggr) \biggr\Vert _{L_{2\pi/r}^{p}}=O_{x} \biggl( ( n+1 ) ^{\beta+\frac{1}{p}+1}A_{n,r}\widetilde{\omega} \biggl( \frac{\pi}{n+1} \biggr) \biggr) . $$

### Corollary 1

*Taking* $r=1$ *the conditions* (11) *and* (13) *in Theorem *1 *reduce to* (1) *and* (2). *Thus we obtain the results from* [2] *and Theorem A* [1, *Theorem *8, *p*. 95], *but in the case of* [3] (*Theorem B and C*) *we reduce the assumptions*.

Next, using more natural conditions when $\beta>0$ we can formulate, without proofs, the following theorems.

### Theorem 4

*Suppose that* $f\in L_{2\pi/r}^{p}$, $1< p<\infty$, $r\in\mathbb{N}$, $0<\beta<1-\frac{1}{p}$. *Let a function* *ω̃* *of the modulus of continuity type satisfy the conditions*:

14 $$ \biggl\{ \int_{\frac{\pi}{r ( n+1 ) }}^{\frac{\pi}{r}} \biggl( \frac{t^{-\gamma} \vert \psi_{x} ( t ) \vert \vert \sin\frac{rt}{2} \vert ^{\beta}}{\widetilde{\omega} ( t ) } \biggr) ^{p}\,dt \biggr\} ^{1/p}=O_{x} \bigl( ( n+1 ) ^{\gamma-\frac{1}{p}} \bigr) $$

*for* $\gamma\in ( \frac{1}{p},\frac{1}{p}+\beta ) $ *and* $r\in \mathbb{N} $ (*instead of* (13)), *and*

$$ \biggl\{ \int_{0}^{\frac{\pi}{r ( n+1 ) }} \biggl( \frac{t \vert \psi_{x} ( t ) \vert \vert \sin\frac{rt}{2}\vert ^{\beta}}{\widetilde{\omega} ( t ) } \biggr) ^{p}\,dt \biggr\} ^{1/p}=O_{x} \bigl( ( n+1 ) ^{-1-\frac{1}{p}} \bigr) $$

*when* $r=1$ *or*

15 $$ \biggl\{ \int_{0}^{\frac{\pi}{r ( n+1 ) }} \biggl( \frac{\vert \psi_{x} ( t ) \vert \vert \sin\frac{rt}{2}\vert ^{\beta}}{\widetilde{\omega} ( t ) } \biggr) ^{p}\,dt \biggr\} ^{1/p}=O_{x} \bigl( ( n+1 ) ^{-\frac{1}{p}} \bigr) $$

*when* $r\geq2$ (*instead of* (11) *and* (12), *respectively*). *If a matrix* *A* *is such that* (9) *and* (8) *are true*, *then*

16 $$ \biggl\vert \widetilde{T}_{n,A} f ( x ) - \widetilde{f_{r}} \biggl( x,\frac{\pi}{r ( n+1 ) } \biggr) \biggr\vert =O_{x} \biggl( ( n+1 ) ^{\beta+1}A_{n,r} \widetilde{\omega} \biggl( \frac{\pi}{n+1} \biggr) \biggr) . $$

*Moreover*, *if a function* *ω̃* *of the modulus of continuity type and a matrix* *A* *satisfy the following conditions*: (14) *with* $r\in \mathbb{N} $ *and* $\gamma\in ( \frac{1}{p},\frac{1}{p}+\beta ) $, (15) *with* $r\in \mathbb{N} $, (9) *and* (10), *then the estimate* (16) *is also true*.

### Theorem 5

*Let* $f\in L_{2\pi/r}^{p} ( \widetilde{\omega} ) _{\beta}$ *with* $1< p<\infty$, $r\in\mathbb{N}$ *and* $0<\beta<1-\frac{1}{p}$. *If a matrix* *A* *is such that* (9) *and* (8) *or* (10) *are true*, *then*

$$ \biggl\Vert \widetilde{T}_{n,A} f ( \cdot) - \widetilde{f}_{r} \biggl( \cdot,\frac{\pi}{r ( n+1 ) } \biggr) \biggr\Vert _{L_{2\pi/r}^{p}}=O_{x} \biggl( ( n+1 ) ^{\beta+1}A_{n,r}\widetilde{\omega} \biggl( \frac{\pi}{n+1} \biggr) \biggr) . $$

### Remark 2

We note that our extra conditions (9), (8) and (10) for a lower triangular infinite matrix $A^{\circ}$ always hold.

### Corollary 2

*Considering the above remarks and the obvious inequality*

17 $$ A_{n,r}\leq rA_{n,1}\quad\textit{for }r\in \mathbb{N} $$

*our results also improve and generalize the mentioned result of Krasniqi* [1].

### Remark 3

We note that instead of $L_{2\pi/r}^{p} ( \widetilde{\omega} ) _{\beta}$ one can consider another subclass of $L_{2\pi/r}^{p}$ generated by any function of the modulus of continuity type e.g. $\widetilde{\omega}_{x}$ such that

$$ \widetilde{\omega}_{x}(f,\delta)=\sup_{ \vert t \vert \leq \delta } \bigl\vert \psi_{x} ( t ) \bigr\vert \leq\widetilde{\omega }_{x} ( \delta) $$

or

$$ \widetilde{\omega}_{x}(f,\delta)=\frac{1}{\delta} \int_{0}^{\delta } \bigl\vert \psi_{x} ( t ) \bigr\vert \,dt\leq\widetilde{\omega}_{x} ( \delta) . $$

## Auxiliary results

We begin this section by some notations from [5] and [4, Sect. 5 of Chapter II]. Let for $r=1,2,\ldots $

$$ D_{k,r}^{\circ} ( t ) =\frac{\sin\frac{ ( 2k+r ) t}{2}}{2\sin\frac{rt}{2}},\qquad \widetilde{D^{\circ}}_{k,r} ( t ) =\frac{\cos\frac{ ( 2k+r ) t}{2}}{2\sin\frac{rt}{2}} $$

and

$$ \widetilde{D}_{k,r} ( t ) =\frac{\cos\frac{rt}{2}-\cos \frac{ ( 2k+r ) t}{2}}{2\sin\frac{rt}{2}}= \frac{\cos\frac{rt}{2}}{2\sin\frac{rt}{2}}-\widetilde{D^{\circ}}_{k,r} ( t ) . $$

It is clear by [4] that

$$ \widetilde{S}_{k}f ( x ) =-\frac{1}{\pi} \int_{-\pi}^{\pi}f ( x+t ) \widetilde{D}_{k,1} ( t ) \,dt $$

and

$$ \widetilde{T}_{n,A} f ( x ) =-\frac{1}{\pi} \int_{-\pi }^{\pi}f ( x+t ) \sum _{k=0}^{\infty}a_{n,k}\widetilde{D}_{k,1} ( t ) \,dt. $$

Now, we present a very useful property of the modulus of continuity.

### Lemma 1

([4])

*A function* *ω̃* *of modulus of continuity type on the interval* $[0,2\pi]$ *satisfies the following condition*:

$$ \delta_{2}^{-1}\widetilde{\omega} ( \delta _{2} ) \leq2\delta_{1}^{-1}\widetilde{\omega} ( \delta_{1} ) \quad\textit{for } \delta_{2}\geq\delta _{1}>0. $$

Next, we present the following well-known estimates.

### Lemma 2

([4])

*If* $0< \vert t \vert \leq\pi$ *then*

$$ \bigl\vert \widetilde{D^{\circ}}_{k,1} ( t ) \bigr\vert \leq \frac{\pi}{2 \vert t \vert }, \qquad\bigl\vert \widetilde{D}_{k,1} ( t ) \bigr\vert \leq\frac{\pi}{ \vert t \vert }$$

*and*, *for any real* *t*, *we have*

$$ \bigl\vert D_{k,1}^{\circ} ( t ) \bigr\vert \leq k+ \frac{1}{2},\qquad \bigl\vert \widetilde{D}_{k,1} ( t ) \bigr\vert \leq\frac{1}{2}k ( k+1 ) \vert t \vert , \qquad \bigl\vert \widetilde{D}_{k,1} ( t ) \bigr\vert \leq k+1. $$

### Lemma 3

([5, 6])

*Let* $r\in N$, $l\in Z$ *and* $(a_{n})\subset \mathbb{C} $. *If* $t \neq \frac{2l\pi}{r}$, *then for every* $m\geq n$

$$\begin{aligned} &\sum_{k=n}^{m}a_{k}\sin kt =-\sum_{k=n}^{m} ( a_{k}-a_{k+r} ) \widetilde{D^{\circ}}_{k,r} ( t ) +\sum _{k=m+1}^{m+r}a_{k}\widetilde{D^{\circ}}_{k,-r} ( t ) -\sum _{k=n}^{n+r-1}a_{k}\widetilde{D^{\circ}}_{k,-r} ( t ) , \\ &\sum_{k=n}^{m}a_{k}\cos kt =\sum_{k=n}^{m} ( a_{k}-a_{k+r} ) D_{k,r}^{\circ} ( t ) -\sum_{k=m+1}^{m+r}a_{k}D_{k,-r}^{\circ } ( t ) +\sum_{k=n}^{n+r-1}a_{k}D_{k,-r}^{\circ} ( t ) . \end{aligned}$$

We additionally need the following estimate as a consequence of Lemma 3.

### Lemma 4

*Let* $r\in \mathbb{N} $, $l\in \mathbb{Z} $ *and* $(a_{n,k})\subset \mathbb{R} _{0}^{+}$ *for* $n.k\in \mathbb{N} _{0}$. *If* $t \neq \frac{2l\pi }{r}$, *then*

$$ \Biggl\vert \frac{1}{2}\sum_{k=0}^{\infty}a_{n,k} \cos\frac{ ( 2k+1 ) t}{2} \Biggr\vert \leq\frac{1}{2 \vert \sin\frac{rt}{2}\vert } \Biggl( A_{n,r}+\sum_{k=0}^{r-1}a_{n,k} \Biggr) \leq\frac{1}{\vert \sin\frac{rt}{2} \vert }A_{n,r}. $$

### Proof

By Lemma 3,

$$\begin{aligned} &\frac{1}{2}\sum_{k=0}^{\infty}a_{n,k} \cos\frac{ ( 2k+1 ) t}{2} \\ &\quad =\frac{1}{2} \Biggl( \sum_{k=0}^{\infty}a_{n,k} \cos kt\cos\frac{t}{2}-\sum_{k=0}^{\infty}a_{n,k} \sin kt\sin\frac{t}{2} \Biggr) \\ &\quad=\frac{\cos\frac{t}{2}}{2} \Biggl( \sum_{k=0}^{\infty} ( a_{n,k}-a_{n,k+r} ) D_{k,r}^{\circ} ( t ) + \sum_{k=0}^{r-1}a_{n,k}D_{k,-r}^{\circ} ( t ) \Biggr) \\ &\qquad{}-\frac{\sin\frac{t}{2}}{2} \Biggl( -\sum_{k=0}^{\infty} ( a_{n,k}-a_{n,k+r} ) \widetilde{D^{\circ}}_{k,r} ( t ) -\sum_{k=0}^{r-1}a_{n,k} \widetilde{D^{\circ}}_{k,-r} ( t ) \Biggr) \end{aligned}$$

and our inequalities follow. □

We also need some special conditions which follow from the ones mentioned above.

### Lemma 5

*Suppose that* $f\in L_{2\pi/r}^{p}$, *where* $1\leq p<\infty$ *and* $r\in \mathbb{N}$. *If the condition* (12) *holds with any function* *ω̃* *of the modulus of continuity type and* $\beta \geq0$, *then*

$$ \biggl\{ \int _{\frac{2 ( m+1 ) \pi}{r}-\frac{\pi}{r ( n+1 ) }}^{\frac{2 ( m+1 ) \pi}{r}} \biggl( \frac{ \vert \psi_{x} ( t ) \vert }{\widetilde{\omega} ( \frac{2 ( m+1 ) \pi}{r}-t ) } \biggr) ^{p} \biggl\vert \sin\frac{rt}{2} \biggr\vert ^{\beta p}\,dt \biggr\} ^{\frac{1}{p}}=O_{x} ( 1 ) , $$

*where* $m\in \{ 0,\ldots [ \frac{r}{2} ] -1 \} $.

### Proof

By the substitution $t=\frac{2 ( m+1 ) \pi}{r}-u$, we obtain

$$\begin{aligned} & \biggl\{ \int_{\frac{2 ( m+1 ) \pi}{r}-\frac{\pi}{r ( n+1 ) }}^{\frac{2 ( m+1 ) \pi}{r}} \biggl( \frac{ \vert \psi_{x} ( t ) \vert }{\widetilde{\omega} ( \frac{2 ( m+1 ) \pi}{r}-t ) } \biggr) ^{p} \biggl\vert \sin\frac{rt}{2} \biggr\vert ^{\beta p}\,dt \biggr\} ^{1/p} \\ &\quad= \biggl\{ \int_{0}^{\frac{\pi}{r ( n+1 ) }} \biggl( \frac{\vert \psi_{x} ( \frac{2 ( m+1 ) \pi}{r}-u ) \vert }{\widetilde{\omega} ( u ) } \biggl\vert \sin\frac{r}{2} \biggl( \frac{2 ( m+1 ) \pi}{r}-u \biggr) \biggr\vert ^{\beta } \biggr) ^{p}\,du \biggr\} ^{1/p} \\ &\quad= \biggl\{ \int_{0}^{\frac{\pi}{r ( n+1 ) }} \biggl( \frac{\vert \psi_{x} ( u ) \vert }{\widetilde{\omega} ( u ) } \biggl\vert \sin\frac{ru}{2} \biggr\vert ^{\beta} \biggr) ^{p}\,du \biggr\} ^{1/p}. \end{aligned}$$

Hence, by (12) our estimate follows. □

### Lemma 6

*Suppose that* $f\in L_{2\pi/r}^{p}$, *where* $1\leq p<\infty$ *and* $r\in \mathbb{N}$. *If the condition* (12) *holds with any function* *ω̃* *of the modulus of continuity type and* $\beta \geq0$, *then*

$$ \biggl\{ \int _{\frac{2m\pi}{r}}^{\frac{2m\pi}{r}+\frac{\pi}{r ( n+1 ) }} \biggl( \frac{ \vert \psi_{x} ( t ) \vert }{\widetilde{\omega} ( t-\frac{2m\pi}{r} ) } \biggr) ^{p} \biggl\vert \sin\frac{rt}{2} \biggr\vert ^{\beta p}\,dt \biggr\} ^{\frac{1}{p}}=O_{x} ( 1 ) , $$

*where* $m\in \{ 0,\ldots [ \frac{r}{2} ] \} $.

### Proof

By the substitution $t=\frac{2m\pi}{r}+u$, analogously to the above proof, we obtain

$$\begin{aligned} & \biggl\{ \int_{\frac{2m\pi}{r}}^{\frac{2m\pi}{r}+\frac{\pi}{r ( n+1 ) }} \biggl( \frac{ \vert \psi_{x} ( t ) \vert }{\widetilde{\omega} ( t-\frac{2m\pi}{r} ) } \biggr) ^{p} \biggl\vert \sin\frac{rt}{2} \biggr\vert ^{\beta p}\,dt \biggr\} ^{1/p} \\ &\quad= \biggl\{ \int_{0}^{\frac{\pi}{r ( n+1 ) }} \biggl( \frac{\vert \psi_{x} ( \frac{2m\pi}{r}+u ) \vert }{\widetilde{\omega} ( u ) } \biggl\vert \sin\frac{r}{2} \biggl( \frac{2m\pi}{r}+u \biggr) \biggr\vert ^{\beta} \biggr) ^{p}\,du \biggr\} ^{1/p} \\ &\quad\leq \biggl\{ \int_{0}^{\frac{\pi}{r ( n+1 ) }} \biggl( \frac{\vert \psi_{x} ( u ) \vert }{\widetilde{\omega} ( u ) } \biggl\vert \sin\frac{ru}{2} \biggr\vert ^{\beta} \biggr) ^{p}\,dt \biggr\} ^{1/p}=O_{x} ( 1 ) \end{aligned}$$

and we have the desired estimate. □

Now, we formulate another two lemmas without proofs. We can prove them in the same way as Lemmas 5 and 6, respectively.

### Lemma 7

*Suppose that* $f\in L_{2\pi/r}^{p}$, *where* $1\leq p<\infty$ *and* $r\in \mathbb{N}$. *If the condition* (13) *holds with any function* *ω̃* *of the modulus of continuity type and* $\gamma ,\beta\geq0$, *then*

$$ \biggl\{ \int_{\frac{2 ( m+1 ) \pi}{r}-\frac{\pi}{r}}^{\frac{2 ( m+1 ) \pi}{r}-\frac{\pi}{r ( n+1 ) }} \biggl( \frac{ \vert \psi_{x} ( t ) \vert \vert \sin\frac{rt}{2} \vert ^{\beta}}{\widetilde{\omega} ( t ) ( \frac{2 ( m+1 ) \pi}{r}-t ) ^{\gamma}} \biggr) ^{p}\,dt \biggr\} ^{1/p}=O_{x} \bigl( ( n+1 ) ^{\gamma} \bigr) , $$

*where* $m\in \{ 0,\ldots [ \frac{r}{2} ] -1 \} $.

### Lemma 8

*Suppose that* $f\in L_{2\pi/r}^{p}$, *where* $1\leq p<\infty$ *and* $r\in \mathbb{N}$. *If the condition* (13) *holds with any function* *ω̃* *of the modulus of continuity type and* $\gamma ,\beta\geq0$, *then*

$$ \biggl\{ \int_{\frac{2m\pi}{r}+\frac{\pi}{r ( n+1 ) }}^{\frac{2m\pi}{r}+\frac{\pi}{r}} \biggl( \frac{ \vert \psi_{x} ( t ) \vert \vert \sin\frac{rt}{2} \vert ^{\beta}}{\widetilde{\omega} ( t ) ( t-\frac{2m\pi}{r} ) ^{\gamma}} \biggr) ^{p}\,dt \biggr\} ^{1/p}=O_{x} \bigl( ( n+1 ) ^{\gamma} \bigr) , $$

*where* $m\in \{ 0,\ldots [ \frac{r}{2} ] \} $.

## Proofs of theorems

### Proof of Theorem 1

It is clear that for odd *r*

$$\begin{aligned} &\widetilde{T}_{n,A} f ( x ) -\widetilde{f}_{r} \biggl( x,\frac{\pi}{r ( n+1 ) } \biggr) \\ &\quad=-\frac{1}{\pi} \int_{0}^{\pi}\psi_{x} ( t ) \sum _{k=0}^{\infty }a_{n,k} \widetilde{D}_{k,1} ( t ) \,dt \\ &\qquad{}+\frac{1}{\pi} \Biggl( \sum_{m=0}^{ [ r/2 ] -1} \int_{\frac{2m\pi}{r}+\frac{\pi}{r ( n+1 ) }}^{\frac{2 ( m+1 ) \pi}{r}-\frac{\pi}{r ( n+1 ) }}+ \int_{\frac{2 [ r/2 ] \pi}{r}+\frac{\pi}{r ( n+1 ) }}^{\frac{ ( 2 [ r/2 ] +1 ) \pi}{r}} \Biggr) \psi_{x} ( t ) \frac{1}{2}\cot\frac{t}{2}\,dt \\ &\quad=-\frac{1}{\pi} \Biggl( \int_{0}^{\frac{\pi}{r ( n+1 ) }}+\sum_{m=1}^{ [ r/2 ] } \int_{\frac{2m\pi}{r}}^{\frac{2m\pi }{r}+\frac{\pi}{r ( n+1 ) }}+\sum_{m=0}^{ [ r/2 ] -1} \int_{\frac{2 ( m+1 ) \pi}{r}-\frac{\pi}{r ( n+1 ) }}^{\frac{2 ( m+1 ) \pi}{r}} \Biggr) \\ &\qquad{}\times\psi_{x} ( t ) \sum_{k=0}^{\infty}a_{n,k} \widetilde{D}_{k,1} ( t ) \,dt \\ &\qquad{}+\frac{1}{\pi} \Biggl( \sum_{m=0}^{ [ r/2 ] } \int_{\frac{2m\pi}{r}+\frac{\pi}{r ( n+1 ) }}^{\frac{2 ( m+1 ) \pi}{r}}+\sum_{m=0}^{[r/2]-1} \int_{\frac{ ( 2m+1 ) \pi}{r}}^{\frac{2 ( m+1 ) \pi}{r}-\frac{\pi}{r ( n+1 ) }} \Biggr) \psi_{x} ( t ) \sum_{k=0}^{\infty}a_{n,k} \widetilde{D^{\circ}}_{k,1} ( t ) \,dt \\ &\quad=I_{0} ( x ) +I_{1} ( x ) +I_{2} ( x ) +I_{3} ( x ) +I_{4} ( x ) \end{aligned}$$

and for even *r*

$$\begin{aligned} &\widetilde{T}_{n,A} f ( x ) -\widetilde{f}_{r} \biggl( x,\frac{\pi}{r ( n+1 ) } \biggr) \\ &\quad=-\frac{1}{\pi} \int_{0}^{\pi}\psi_{x} ( t ) \sum _{k=0}^{\infty }a_{n,k} \widetilde{D}_{k,1} ( t ) \,dt+\frac{1}{\pi}\sum _{m=0}^{ [ r/2 ] -1} \int_{\frac{2m\pi}{r}+\frac{\pi}{r ( n+1 ) }}^{\frac{2 ( m+1 ) \pi}{r}-\frac{\pi}{r ( n+1 ) }}\psi_{x} ( t ) \frac{1}{2}\cot\frac{t}{2}\,dt \\ &\quad=-\frac{1}{\pi} \Biggl( \int_{0}^{\frac{\pi}{r ( n+1 ) }}+\sum_{m=1}^{ [ r/2 ] -1} \int_{\frac{2m\pi}{r}}^{\frac{2m\pi}{r}+\frac{\pi}{r ( n+1 ) }}+\sum_{m=0}^{ [ r/2] -1} \int_{\frac{2 ( m+1 ) \pi}{r}-\frac{\pi}{r ( n+1 ) }}^{\frac{2 ( m+1 ) \pi}{r}} \Biggr) \\ &\qquad{}\times\psi_{x} ( t ) \sum_{k=0}^{\infty}a_{n,k} \widetilde{D}_{k,1} ( t ) \,dt \\ &\qquad{}+\frac{1}{\pi} \Biggl( \sum_{m=0}^{ [ r/2 ] -1} \int_{\frac{2m\pi}{r}+\frac{\pi}{r ( n+1 ) }}^{\frac{ ( 2m+1 ) \pi}{r}}+\sum_{m=0}^{ [ r/2 ] -1} \int_{\frac{ ( 2m+1 ) \pi}{r}}^{\frac{2 ( m+1 ) \pi}{r}-\frac{\pi}{r ( n+1 ) }} \Biggr) \\ &\qquad{}\times\psi_{x} ( t ) \sum_{k=0}^{\infty}a_{n,k} \widetilde{D^{\circ}}_{k,1} ( t ) \,dt \\ &\quad=I_{0} ( x ) +I_{1}^{\prime} ( x ) +I_{2} ( x ) +I_{3}^{\prime} ( x ) +I_{4} ( x ) , \end{aligned}$$

whence

$$\begin{aligned} & \biggl\vert \widetilde{T}_{n,A} f ( x ) - \widetilde{f}_{r} \biggl( x,\frac{\pi}{r ( n+1 ) } \biggr) \biggr\vert \\ &\quad \leq \bigl\vert I_{0} ( x ) \bigr\vert + \bigl\vert I_{1} ( x ) \bigr\vert + \bigl\vert I_{1}^{\prime} ( x ) \bigr\vert + \bigl\vert I_{2} ( x ) \bigr\vert + \bigl\vert I_{3} ( x ) \bigr\vert + \bigl\vert I_{3}^{\prime} ( x ) \bigr\vert + \bigl\vert I_{4} ( x ) \bigr\vert . \end{aligned}$$

Next, using Lemma 2, (8), the Hölder inequality with $p>1$ and $q=\frac{p}{p-1}$ and (11) when $r=1$ or (12) when $r\geq2$ we get

$$\begin{aligned} & \bigl\vert I_{0} ( x ) \bigr\vert \\ &\quad=O \bigl( ( n+1 ) ^{2} \bigr) \int_{0}^{\frac{\pi}{r ( n+1 ) }}t \bigl\vert \psi_{x} ( t ) \bigr\vert \,dt \\ &\quad\leq O \bigl( ( n+1 ) ^{2} \bigr) \biggl\{ \int_{0}^{\frac{\pi}{r ( n+1 ) }} \biggl( \frac{t \vert \psi_{x} ( t ) \vert }{\widetilde{\omega} ( t ) } \biggr) ^{p}\sin^{\beta p}\frac{rt}{2}\,dt \biggr\} ^{1/p} \biggl\{ \int_{0}^{\frac{\pi}{r ( n+1 ) }} \biggl( \frac{\widetilde{\omega} ( t ) }{\sin^{\beta}\frac{rt}{2}} \biggr) ^{q}\,dt \biggr\} ^{\frac{1}{q}} \\ &\quad\leq O \bigl( ( n+1 ) ^{2} \bigr) O_{x} \bigl( ( n+1 ) ^{-1} \bigr) \widetilde{\omega} \biggl( \frac{\pi}{r ( n+1 ) } \biggr) \biggl\{ \int_{0}^{\frac{\pi}{r ( n+1 ) }} \biggl( \frac{\pi }{rt} \biggr) ^{\beta q}\,dt \biggr\} ^{\frac{1}{q}} \\ &\quad=O_{x} \bigl( ( n+1 ) \bigr) \widetilde{\omega} \biggl( \frac{\pi }{r ( n+1 ) } \biggr) \biggl( \frac{\pi}{r ( n+1 ) } \biggr) ^{\frac {1}{q}-\beta}=O_{x} \bigl( ( n+1 ) ^{\beta+\frac{1}{p}} \bigr) \widetilde{\omega} \biggl( \frac{\pi}{n+1} \biggr) , \end{aligned}$$

for $0\leq\beta<1-\frac{1}{p}$. We note that applying the condition (9) we have

$$\begin{aligned} \bigl[ ( n+1 ) A_{n,r} \bigr] ^{-1}&= \Biggl[ \sum _{l=0}^{n}A_{n,r} \Biggr] ^{-1}\leq \Biggl[ \sum_{l=0}^{n} \sum_{k=l}^{\infty} \vert a_{n,k}-a_{n,k+r} \vert \Biggr] ^{-1} \\ &\leq \Biggl[ \sum_{l=0}^{n} \Biggl\vert \sum_{k=l}^{\infty} ( a_{n,k}-a_{n,k+r} ) \Biggr\vert \Biggr] ^{-1}= \Biggl[ \sum _{l=0}^{n}\sum _{k=l}^{r+l-1}a_{n,k} \Biggr] ^{-1}=O ( 1 ) , \end{aligned}$$

whence

$$ \bigl\vert I_{0} ( x ) \bigr\vert =O_{x} \biggl( ( n+1 ) ^{1+\beta+\frac{1}{p}}A_{n,r}\widetilde{\omega} \biggl( \frac{\pi}{n+1} \biggr) \biggr) . $$

By Lemma 2

$$\begin{aligned} & \bigl\vert I_{1} ( x ) \bigr\vert + \bigl\vert I_{1}^{\prime } ( x ) \bigr\vert + \bigl\vert I_{2} ( x ) \bigr\vert \\ &\quad\leq \frac{1}{\pi} \Biggl( \sum_{m=1}^{ [ r/2 ] } \int_{\frac{2m\pi}{r}}^{\frac{2m\pi}{r}+\frac{\pi}{r ( n+1 ) }}+\sum_{m=0}^{ [ r/2 ] -1} \int_{\frac{2 ( m+1 ) \pi }{r}-\frac{\pi}{r ( n+1 ) }}^{\frac{2 ( m+1 ) \pi}{r}} \Biggr) \frac{ \vert \psi_{x} ( t ) \vert }{t}\,dt \\ &\quad \leq\frac{1}{\pi} \Biggl( \sum_{m=1}^{ [ r/2 ] } \int_{\frac{2m\pi}{r}}^{\frac{2m\pi}{r}+\frac{\pi}{r ( n+1 ) }}+\sum_{m=0}^{ [ r/2 ] -1} \int_{\frac{2 ( m+1 ) \pi }{r}-\frac{\pi}{r ( n+1 ) }}^{\frac{2 ( m+1 ) \pi}{r}} \Biggr) \frac{ \vert \psi_{x} ( t ) \vert }{\pi/r}\,dt \end{aligned}$$

and using the Hölder inequality with $p>1$ and $q=\frac{p}{p-1}$

$$\begin{aligned} & \bigl\vert I_{1} ( x ) \bigr\vert + \bigl\vert I_{1}^{\prime } ( x ) \bigr\vert + \bigl\vert I_{2} ( x ) \bigr\vert \\ &\quad \leq O_{x} ( 1 ) \sum_{m=1}^{ [ r/2 ] } \biggl[ \int_{\frac{2m\pi}{r}}^{\frac{2m\pi}{r}+\frac{\pi}{r ( n+1 ) }} \biggl( \frac{ \vert \psi_{x} ( t ) \vert \sin^{\beta}\frac{rt}{2}}{\widetilde{\omega} ( t-\frac{2m\pi}{r} ) } \biggr) ^{p}\,dt \biggr] ^{\frac{1}{p}} \\ &\qquad{}\times \biggl[ \int_{\frac{2m\pi}{r}}^{\frac{2m\pi}{r}+\frac{\pi}{r ( n+1 ) }} \biggl( \frac{\widetilde{\omega} ( t-\frac{2m\pi}{r}) }{\sin^{\beta}\frac{rt}{2}} \biggr) ^{q}\,dt \biggr] ^{\frac{1}{q}} \\ &\qquad{}+O_{x} ( 1 ) \sum_{m=1}^{ [ r/2 ] -1} \biggl[ \int_{\frac{2 ( m+1 ) \pi}{r}-\frac{\pi}{r ( n+1 ) }}^{\frac{2 ( m+1 ) \pi}{r}} \biggl( \frac{ \vert \psi_{x} ( t ) \vert \sin^{\beta}\frac{rt}{2}}{\widetilde{\omega} ( \frac{2 ( m+1 ) \pi}{r}-t ) } \biggr) ^{p}\,dt \biggr] ^{\frac{1}{p}} \\ &\qquad{}\times \biggl[ \int_{\frac{2 ( m+1 ) \pi}{r}-\frac{\pi}{r ( n+1 ) }}^{\frac{2 ( m+1 ) \pi}{r}} \biggl( \frac{\widetilde{\omega} ( \frac{2 ( m+1 ) \pi}{r}-t ) }{\sin^{\beta}\frac{rt}{2}} \biggr) ^{q}\,dt \biggr] ^{\frac{1}{q}}. \end{aligned}$$

Hence, by Lemmas 5 and 6 with (12) and (9),

$$\begin{aligned} &\bigl\vert I_{1} ( x ) \bigr\vert + \bigl\vert I_{1}^{\prime } ( x ) \bigr\vert + \bigl\vert I_{2} ( x ) \bigr\vert \\ &\quad=O_{x} ( 1 ) \widetilde{\omega} \biggl( \frac{\pi}{r ( n+1 ) } \biggr) \biggl[ \int_{0}^{\frac{\pi}{r ( n+1 ) }} \biggl( \frac{1}{\sin^{\beta}\frac {rt}{2}} \biggr) ^{q}\,dt \biggr] ^{\frac{1}{q}} \\ &\quad =O_{x} \bigl( ( n+1 ) ^{\beta-\frac{1}{q}} \bigr) \widetilde{\omega} \biggl( \frac{\pi}{n+1} \biggr) =O_{x} \biggl( ( n+1 ) ^{\beta+\frac{1}{p}}A_{n,r}\widetilde{\omega} \biggl( \frac{\pi}{n+1} \biggr) \biggr) , \end{aligned}$$

for $0\leq\beta<1-\frac{1}{p}$.

In the case of the last integrals, applying Lemma 4 we obtain

$$\begin{aligned} & \bigl\vert I_{3} ( x ) \bigr\vert + \bigl\vert I_{3}^{\prime } ( x ) \bigr\vert + \bigl\vert I_{4} ( x ) \bigr\vert \\ &\quad\leq\frac{1}{\pi} \Biggl( \sum_{m=0}^{ [ r/2 ] } \int_{\frac{2m\pi}{r}+\frac{\pi}{r ( n+1 ) }}^{\frac{ ( 2m+1 ) \pi}{r}}+\sum_{m=0}^{ [ r/2 ] -1} \int_{\frac{ ( 2m+1 ) \pi}{r}}^{\frac{2 ( m+1 ) \pi}{r}-\frac{\pi}{r ( n+1 ) }} \Biggr) \frac{ \vert \psi_{x} ( t ) \vert }{\vert \sin\frac{t}{2}\sin\frac{rt}{2} \vert }A_{n,r}\,dt. \end{aligned}$$

Using the estimates $\vert \sin\frac{t}{2} \vert \geq\frac{\vert t \vert }{\pi}$ for $t\in [ 0,\pi ] $, $\vert \sin\frac{rt}{2} \vert \geq\frac{rt}{\pi}-2m$ for $t\in [ \frac{2m\pi}{r}+\frac{\pi}{r ( n+1 ) },\frac{ ( 2m+1 ) \pi}{r} ] $, where $m\in \{ 0,\ldots, [ r/2 ] \} $ and $\vert \sin\frac{rt}{2} \vert \geq2 ( m+1 ) -\frac{rt}{\pi}$ for $t\in [ \frac{ ( 2m+1 ) \pi}{r},\frac{2 ( m+1 ) \pi}{r}-\frac{\pi}{r ( n+1 ) } ] $, where $m\in \{ 0,\ldots, [ r/2 ] -1 \} $, we obtain

$$\begin{aligned} & \bigl\vert I_{3} ( x ) \bigr\vert + \bigl\vert I_{3}^{\prime } ( x ) \bigr\vert + \bigl\vert I_{4} ( x ) \bigr\vert \\ &\quad\leq A_{n,r}\sum_{m=0}^{ [ r/2 ] } \int_{\frac{2m\pi}{r}+\frac{\pi}{r ( n+1 ) }}^{\frac{ ( 2m+1 ) \pi}{r}}\frac{\vert \psi_{x} ( t ) \vert }{\frac{rt}{\pi} ( t-\frac{2m\pi}{r} ) }\,dt \\ &\qquad{}+A_{n,r}\sum_{m=0}^{ [ r/2 ] -1} \int_{\frac{ ( 2m+1 ) \pi}{r}}^{\frac{2 ( m+1 ) \pi}{r}-\frac{\pi}{r ( n+1 ) }}\frac{ \vert \psi_{x} ( t ) \vert }{\frac{rt}{\pi} [ \frac{2 ( m+1 ) \pi}{r}-t ] }\,dt. \end{aligned}$$

By the Hölder inequality with $p>1$ and $q=\frac{p}{p-1}$ we have

$$\begin{aligned} & \bigl\vert I_{3} ( x ) \bigr\vert + \bigl\vert I_{3}^{\prime } ( x ) \bigr\vert + \bigl\vert I_{4} ( x ) \bigr\vert \\ &\quad\leq\frac{\pi}{r}A_{n,r}\sum_{m=0}^{ [ r/2 ] } \biggl[ \int _{\frac{2m\pi}{r}+\frac{\pi}{r ( n+1 ) }}^{\frac{2m\pi }{r}+\frac{\pi}{r}} \biggl( \frac{ \vert \psi_{x} ( t ) \vert }{\widetilde{\omega} ( t ) ( t-\frac{2m\pi}{r}) ^{\gamma}} \biggl\vert \sin\frac{rt}{2} \biggr\vert ^{\beta} \biggr) ^{p}\,dt \biggr] ^{\frac{1}{p}} \\ &\qquad{}\times \biggl[ \int _{\frac{2m\pi}{r}+\frac{\pi}{r ( n+1 ) }}^{\frac{2m\pi}{r}+\frac{\pi}{r}} \biggl( \frac{\widetilde{\omega} ( t ) ( t-\frac{2m\pi}{r} ) ^{\gamma}}{t ( t-\frac{2m\pi }{r} ) \vert \sin\frac{rt}{2} \vert ^{\beta}} \biggr) ^{q}\,dt \biggr] ^{\frac{1}{q}} \\ &\qquad{}+\frac{\pi}{r}A_{n,r}\sum_{m=0}^{ [ r/2 ] -1} \biggl[ \int _{\frac{2 ( m+1 ) \pi}{r}-\frac{\pi}{r}}^{\frac{2 ( m+1 ) \pi}{r}-\frac{\pi}{r ( n+1 ) }} \biggl( \frac{ \vert \psi _{x} ( t ) \vert }{\widetilde{\omega} ( t ) ( \frac{2 ( m+1 ) \pi}{r}-t ) ^{\gamma}} \biggl\vert \sin\frac{rt}{2} \biggr\vert ^{\beta} \biggr) ^{p}\,dt \biggr] ^{\frac{1}{p}} \\ &\qquad{}\times\biggl[ \int _{\frac{2 ( m+1 ) \pi}{r}-\frac{\pi}{r}}^{\frac{2 ( m+1 ) \pi}{r}-\frac{\pi}{r ( n+1 ) }} \biggl( \frac {\widetilde{\omega} ( t ) ( \frac{2 ( m+1 ) \pi }{r}-t ) ^{\gamma}}{t ( \frac{2 ( m+1 ) \pi}{r}-t ) \vert \sin\frac{rt}{2} \vert ^{\beta}} \biggr) ^{q}\,dt \biggr] ^{\frac{1}{q}}. \end{aligned}$$

Further, using Lemmas 7 and 8 with (13) and Lemma 1 we get

$$\begin{aligned} & \bigl\vert I_{3} ( x ) \bigr\vert + \bigl\vert I_{3}^{\prime } ( x ) \bigr\vert + \bigl\vert I_{4} ( x ) \bigr\vert \\ &\quad\leq O_{x} ( 1 ) A_{n,r}\sum _{m=0}^{ [ r/2 ] } ( n+1 ) ^{\gamma} \biggl[ \int _{\frac{2m\pi}{r}+\frac{\pi}{r ( n+1 ) }}^{\frac{2m\pi}{r}+\frac{\pi}{r}} \biggl( \frac{\widetilde{\omega} ( t ) ( t-\frac{2m\pi}{r} ) ^{\gamma }}{t ( t-\frac{2m\pi}{r} ) \vert \sin\frac{rt}{2} \vert ^{\beta}} \biggr) ^{q}\,dt \biggr] ^{\frac{1}{q}} \\ &\qquad{}+O_{x} ( 1 ) A_{n,r}\sum_{m=0}^{ [ r/2 ] -1} ( n+1 ) ^{\gamma} \biggl[ \int _{\frac{2 ( m+1 ) \pi}{r}-\frac{\pi}{r}}^{\frac{2 ( m+1 ) \pi}{r}-\frac{\pi}{r ( n+1 ) }} \biggl( \frac{\widetilde{\omega} ( t ) ( \frac{2 ( m+1 ) \pi}{r}-t ) ^{\gamma}}{t ( \frac{2 ( m+1 ) \pi}{r}-t ) \vert \sin\frac{rt}{2} \vert ^{\beta }} \biggr) ^{q}\,dt \biggr] ^{\frac{1}{q}} \\ &\quad= O_{x} ( 1 ) A_{n,r} \Biggl[ \sum _{m=0}^{ [ r/2 ] } ( n+1 ) ^{\gamma} \biggl\{ \int _{\frac{\pi}{r(n+1)}}^{\frac{\pi}{r}} \biggl( \frac{\widetilde{\omega} ( t+\frac{2m\pi}{r} ) t^{\gamma -1}}{ ( t+\frac{2m\pi}{r} ) \vert \sin\frac{rt}{2}\vert ^{\beta}} \biggr) ^{q}\,dt \biggr\} ^{\frac{1}{q}} \\ &\qquad{}+ \sum_{m=0}^{ [ r/2 ] -1} ( n+1 ) ^{\gamma } \biggl\{ \int _{\frac{\pi}{r(n+1)}}^{\frac{\pi}{r}} \biggl( \frac{\widetilde{\omega} ( \frac{2(m+1)\pi}{r}-t ) t^{\gamma-1}}{( \frac{2(m+1)\pi}{r}-t ) \vert \sin\frac{rt}{2} \vert ^{\beta}} \biggr) ^{q}\,dt \biggr\} ^{\frac{1}{q}} \Biggr] \\ &\quad=O_{x} ( 1 ) A_{n,r} ( n+1 ) ^{\gamma} \biggl\{ \int _{\frac{\pi}{r(n+1)}}^{\frac{\pi}{r}} \biggl( \frac{\widetilde{\omega} ( t ) t^{\gamma-1}}{t \vert \sin\frac{rt}{2}\vert ^{\beta}} \biggr) ^{q}\,dt \biggr\} ^{\frac{1}{q}} \\ &\quad=O_{x} ( 1 ) A_{n,r} ( n+1 ) ^{1+\gamma} \widetilde{\omega} \biggl( \frac{\pi}{r ( n+1 ) } \biggr) \biggl( \int _{\frac{\pi}{r(n+1)}}^{\frac{\pi}{r}}t^{ ( \gamma-1-\beta ) q}\,dt \biggr) ^{\frac{1}{q}} \\ &\quad =O_{x} ( 1 ) A_{n,r} ( n+1 ) ^{1+\gamma} \widetilde{\omega} \biggl( \frac{\pi}{r ( n+1 ) } \biggr) ( n+1 ) ^{1+\beta-\gamma-\frac{1}{q}} \\ &\quad=O_{x} \biggl( ( n+1 ) ^{1+\beta+\frac{1}{p}}A_{n,r} \widetilde{\omega} \biggl( \frac{\pi}{ ( n+1 ) } \biggr) \biggr) \end{aligned}$$

for $0<\gamma<\beta+\frac{1}{p}$.

Collecting the partial estimates our statement follows.

### Proof of Theorem 2

The proof is the same as above, but for estimate of $\vert I_{0} ( x ) \vert $ we only used the inequality $\vert \widetilde{D}_{k,1} ( t ) \vert \leq k+1$ from Lemma 2, and the condition (10) instead of (8).

### Proof of Theorem 3

We note that for the estimate of $\Vert \widetilde{T}_{n,A} f ( \cdot ) -\widetilde{f}_{r} ( \cdot,\frac{\pi}{(n+1)}) \Vert _{L_{2\pi}^{p}}$ we need the conditions on *ω̃* from the assumptions of Theorems 1 or 2. These conditions always hold with $\Vert \psi_{\cdot} ( t ) \Vert _{L_{2\pi /r}^{p}} $ instead of $\vert \psi_{x} ( t ) \vert $ and thus the desired result follows.
